# Prospects and challenges for computer simulations of monolayer-protected metal clusters

**DOI:** 10.1038/s41467-021-22545-x

**Published:** 2021-04-13

**Authors:** Sami Malola, Hannu Häkkinen

**Affiliations:** 1 Department of Physics, Nanoscience Center, University of Jyväskylä, Jyväskylä, Finland; 2 Department of Chemistry, Nanoscience Center, University of Jyväskylä, Jyväskylä, Finland

**Keywords:** Computational chemistry, Nanoparticles

## Abstract

Precise knowledge of chemical composition and atomic structure of functional nanosized systems, such as metal clusters stabilized by an organic molecular layer, allows for detailed computational work to investigate structure-property relations. Here, we discuss selected recent examples of computational work that has advanced understanding of how these clusters work in catalysis, how they interact with biological systems, and how they can make self-assembled, macroscopic materials. A growing challenge is to develop effective new simulation methods that take into account the cluster-environment interactions. These new hybrid methods are likely to contain components from electronic structure theory combined with machine learning algorithms for accelerated evaluations of atom-atom interactions.

Monolayer-protected metal clusters (MPCs) are hybrid metal nanoparticles consisting of a metal core and a protecting layer of organic ligand molecules. They have a precise mass and chemical composition, and in many cases their structure is known to atomic precision^1^. During the last decade, experimental and computational investigations of MPCs have burgeoned, yielding ample novel information about physical, chemical, catalytic, optical, biological and medical properties of these atomically defined nanomaterials^1^. The often precise knowledge of the MPCs’ atomic structure creates an excellent starting point to use various atomistic simulation tools to understand their structure-property relations. However, MPCs can have complex interactions with their environments, which creates challenges to simulations due to needed length scales, time scales or the need to describe the complex chemical interactions properly on an equal footing.

As Fig. 1 illustrates, there are currently several methods that can be used for computational investigations of MPCs’ physical and chemical properties. Quantum-chemical (QC) methods are based on optimizing the total many-body wave function of the quantum system (a function of 3N components for N atoms). They can conveniently deal with small molecules with great chemical accuracy and have traditionally provided benchmark results for strengths of various chemical bonds. However, their application area is still limited to small systems on the order of 10 metal atoms. Density functional theory (DFT) is a major workhorse dealing with MPCs’ electronic structure, optical properties and reactions. DFT uses the total electron density of the system (a function of 3 components) as the basic physical variable, reducing the complexity of the ground-state problem immensely as compared to QC methods. With modern massively parallel supercomputers, the most efficient DFT codes can be used to optimize atomic structures and calculate ground-state properties of systems of up to a thousand atoms or more. An important extension of the ground-state DFT method, the so-called time-dependent DFT, allows for calculations of excited-state properties as well. Dynamical simulations using DFT forces are however limited to rather short time scales, on the order of 100 ps. In this time scale, breaking and forming of chemical bonds, as well as structural transformation of small nanoparticles could be studied. To reach the size and time scales beyond the limits of DFT, there is a set of widely available and well-known methods based on pre-parametrized atomistic interaction potentials or “force fields”. They allow for structural optimization (molecular mechanics, MM) or extended dynamical simulations (molecular dynamics, MD) of atomistic nanostructures up to millions of atoms and up to microsecond time scales. Finally, beyond the atomistic spatial resolution, components in complex nanostructures can be defined as “unified atoms” in coarse-grained (CG) methods, and dynamics of the system are described by interactions between the unified atoms instead of real atoms. This simplification allows for dynamical simulations of mesoscale materials up to millisecond time scale.

**Fig. 1:**
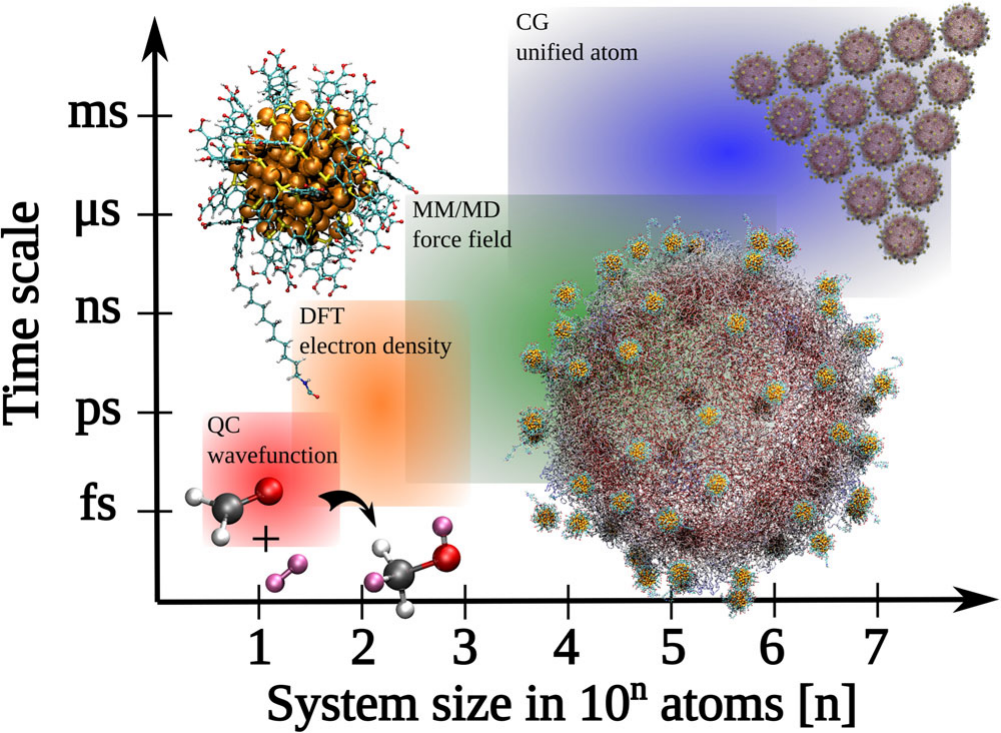
System size vs. time scales and relevant simulation methods from molecules to self-assembled nanostructures. Quantum-chemical (QC) methods can conveniently deal with small molecules with great accuracy. Density functional theory (DFT) is a major workhorse dealing with MPCs’ electronic structure, optical properties, and reactions, but with limited time scales. Classical force fields generated for molecular mechanics (MM) and molecular dynamics (MD) simulations can give valuable details on MPC-environment interactions such as with viruses as shown here. Finally, processes and properties in self-assembling MPC systems may need to be dealt with coarse-grained (CG) methods where each MPC is described as a “unified atom”.

Here we discuss some of the recent advances where simulations, tightly connected to experiments, have been able to reveal details of MPC functionality related to catalysis, their interactions with biological objects, and self-assembly into cluster-based materials. In these areas, considered as promising applications of MPCs, efficient and reliable simulation methods are needed to take cluster-environment effects properly into account. We finish by briefly discussing future prospects to speed up atomic-scale simulations of cluster structures and complex cluster-environment interactions.

## Catalysis

Metal nanoparticles have played an important role in catalysis for more than 100 years. Over that time, Nobel-winning innovations have facilitated development of a range of nanoparticle-based catalysts that work from cleaning automotive exhaust to producing fertilizers in order to secure food for the ever-growing global population. Much of this development has been “trial-and-error” engineering. MPCs can, in principle, aid rational design of new nanostructured catalysts, since they allow for a chemically scalable way to produce model catalysts where each catalytic particle is of the same size and structure. This can help to correlate catalytic behavior to the catalyst’s size and structure. Many sub-areas of catalysis are likely to benefit from MPCs: their use in homogeneous and organic catalysis may offer new concepts for production of drugs and fine chemicals, and they may be promising in electrocatalysis for reactions such as hydrogen production and CO_2_ capture/reduction. In all of these areas, it will be challenging to map in detail the atomistic reaction paths and mechanisms. Discussed below are some of the first cases where simulations have been able to help considerably the interpretation of experimental results.

Catalytic reactions involve making and breaking chemical bonds by electron transfer, which usually requires the reactant molecules to be in chemical contact with the catalytic metal. This raises a fundamental question about whether the protecting ligand layer of MPCs can inhibit the reactions by “poisoning” the catalyst. Zheng’s group has recently demonstrated^2^ that the atomically precise copper-hydride cluster Cu_25_H_10_(SPhCl_2_)_18_^3-^ can catalyze ketone hydrogenation in homogeneous phase in mild conditions, and accompanying DFT calculations together with NMR data of reaction intermediates were able to reveal detailed mechanistic pathways for the reaction. Simulations showed that despite the protecting thiolate layer, there is enough solvent accessibility for the reactants to reach the core-ligand interface. Furthermore, simulations revealed that the copper-hydride core acts as the hydrogen source in the reaction.

MPCs have been used for a decade or so as model systems also in heterogeneous catalysis, either at chemically inert (such as amorphous carbon) or active (such as reducible oxides) supports. In all cases, the supported MPCs usually need to be “activated” before reactions by heat treatment, which may modify the atomic and electronic structure of the MPCs. Lack of detailed information about the effects of activation has made it very challenging to model the catalytic reactions. Recently, work by the groups of Donoeva^3^ and Barrabes^4^ have provided important insight into cluster-support interactions during activation. Donoeva et al. showed^3^ that the basicity/acidity of the support oxide (ability to transfer electrons to/from the cluster) can lead to two drastically different behaviors of phosphine-stabilized gold clusters during activation. On basic ceria support, the phosphine layer can be “peeled off” leaving an exposed gold nanocluster, while a total fragmentation of the cluster was observed on acidic silica support. DFT calculations quantified the corresponding thermodynamic driving forces for these processes via Born-Haber cycle. Barrabes et al. showed^4^ a similar “peeling off” or migration of thiolate ligands from a gold cluster on ceria and alumina supports during activation. These examples demonstrate that DFT simulations of any reactions catalyzed by MPCs on oxides need to deal with a high degree of complexity regarding the size and the composition of the unit cell, ideally incorporating both the naked cluster and the ligands on the support to account for the secondary effects of the proximal ligands on the electronic structure of the cluster and the oxide.

## MPC–bio interactions

There is an increasing need to understand interactions and functionalities of MPCs in biological environments due to the large potential of using nanometer-sized particles in imaging, sensing, tracking of biomolecules inside cells, and drug delivery in nanomedicine^5^. The biochemical environment is complex, consisting of a variety of salt concentrations, pH changes, and external biomolecules interacting with the organic MPC surface for rather long diffusion-controlled timescales. Here, computational approaches are expected to gain significance in future research, since they can provide detailed molecular-level information of changes in surface chemistry during the MPC-bio-interaction time, which would help to design optimally bio-compatible MPCs^6^. However, none of the currently available methods (Fig. 1) can alone handle simulations of full processes, such as efforts to understand long-term stability of MPCs exposed to bio-thiols (such as cysteine and glutathione) or formation of protein corona around MPCs.

Our group has had a long-term interest to develop force field models that allow classical MD simulations of MPCs in biological environments^7^. Using the force fields, MD simulations can extend the physical time scale of phenomena to be studied up to microsecond range (Fig. 1), allowing, for example, for investigations of the dynamics of the ligand layer in solution, or rather reliable estimates of binding affinity of ligand-functionalized MPCs to biomolecules and biological objects such as viruses. We have used a combination of DFT calculations and MD simulations to decipher the hugely complicated ^1^H NMR spectrum of the Au_102_(pMBA)_44_ clusters (pMBA = para mercaptobenzoic acid) measured in water, where MD simulations interestingly revealed also very anisotropic ligand dynamics^8^. Million-atom MD simulations were able to estimate the binding affinities of functionalized gold-based MPCs to enteroviruses^9^, which helped to interpret NMR and electron microscopy (EM) data of virus-MPC complexes^10^. Gold-based MPCs have been demonstrated as contrast agents in high-resolution EM imaging of viruses^11^, but they may potentially also be used for manipulation of the virus capsid dynamics and detection of the released RNA/DNA, generating crucial information about molecule-level events triggering virus infection inside cells. Successful simulations to address these and other grand challenges related to functionality of MPCs in biological environments need to utilize hybrid methods that can bridge the domains of quantum mechanics (QM) and classical molecular mechanics (MM)/molecular dynamics methods. Optimizing the QM/MM methods to effectively treat systems comprising millions of atoms and take advantage of the new generations of supercomputers will be needed.

## Self-assembly and cluster-based materials

Ability to design nanomaterials “at will” with desired structures and functionalities has been one of the fundamental driving forces in nanoscience since the days of Feynman. MPCs in principle offer vast possibilities for such design since the currently known stable MPCs vary widely in their solubility and structural, electronic, and optical properties, from aqueous to organic phase, from non-metallic (molecule-like) to metallic (plasmonic), from non-fluorescent to bright fluorescent, to name a few. A major challenge is to decipher efficient self-assembling mechanisms by which macroscopic cluster-based materials could be made, and to correlate the properties of the materials to their individual building blocks. Atomistic simulations can greatly help in this challenge, since they can uncover detailed correlations between atom-scale variations and properties of individual clusters, and help to decipher optimal interactions between clusters for self-assembly. Two recent examples are discussed here.

Nonappa et al. discovered^12^ an interesting spontaneous self-assembly of Au_102_(pMBA)_44_ clusters into 2D flakes and 3D spherical capsids in water-methanol mixtures. The superstructures were up to 500 nm in size and could be reversibly assembled and de-assembled by controlling the solvent conditions. Preliminary MD simulations done in our group^13^ probed for the stability of superstructures of model 3D capsids consisting of 100 Au_102_(pMBA)_44_ clusters. We varied the composition of the solvent as well as the protonation/deprotonation states of the pMBA ligands, studying the dynamics of the systems up to 30 ns after equilibration stage. The MD simulations showed, intriguingly, that the most stable capsids were the ones enclosing water inside and methanol outside the capsid. Additional stabilizing effects from chelating counterions were found. This shows the potential of all-atom classical MD simulations for investigations of cluster-environment interactions that are relevant for superstructures’ self-assembly.

Zheng et al.^14^ fabricated macroscopic (up to about 0.5 mm) needle-like single crystals consisting of polymeric cluster material where the building blocks were intermetallic 34-atom gold-silver clusters. The clusters were passivated by bulky 1-ethynyladamantane molecules which acted as electrically insulating material in the cross-directions of the polymer, yielding 1800-fold difference in *p*-type conductivity along and across the polymer axis. DFT computations determined the fundamental band gap of about 1.3 eV for this cluster material, confirming the measured semiconducting behavior in a field-effect-transistor device. Further DFT simulations on this and other similar systems could help to investigate in detail how variations in the size and intermetallic composition of the clusters and their linking motifs will affect the conductivity, providing useful information for future experiments to optimize the structure of the system for desired properties, such as the semiconducting band gap or degree of conductivity.

## Prospects for machine learning

Machine learning and data-driven methods have rapidly gained popularity for analysis of structure-property correlations in materials science^15^. A pragmatic view on ML methods considers them as highly sophisticated, non-linear fitting schemes of multi-dimensional data. Given enough data points to allow for extrapolation or interpolation of an object result, an algorithm can “learn” to predict a new result (target property) when it operates within source information that is sufficiently within limits of the training data.

One of the great promises of ML methods in material science lies in their demonstrated ability to reproduce energies and forces of atom-atom interactions in a number of systems, provided that sufficient amount of reference data has been provided for training. Usually, the training data is obtained from higher-lever DFT calculations or MD simulations using DFT forces. The majority of demonstrated successful cases have been either bulk materials or very small molecules. MPCs are challenging systems since many times they lack high-symmetry atom structure and the range of forces cover basically the full scale of metallic, ionic, covalent, and non-covalent weak interactions. Therefore, one has to rely on DFT to produce the training data for fitting the ML interatomic potentials, which makes that necessary step very demanding numerically.

Our group has recently demonstrated that it is indeed possible to model the structure and dynamics of a realistic MPC, namely the well-known Au_38_(SR)_24_ nanocluster^16^. Instead of the popular neural networks, we chose to use distance-based ML methods in fitting the interatomic potential employing the so-called (Extreme) Minimal Learning Machine (E)MLM strategy^17,18^. The training data was collected from our previously published DFT MD simulations for two known structural isomers of the Au_38_(SR)_24_ cluster^19^. The resulting ML potential was used in Monte Carlo simulations of the dynamics of cluster isomers and was demonstrated to reproduce the structural parameters and energetics at several temperatures with a reasonable accuracy compared to reference DFT MD data while speeding up the simulations by several orders of magnitude. These results open up way for further developments for efficient atomistic simulations of MPCs retaining close-to DFT accuracy, which could be used for predictions of yet-unknown MPC compositions structures^20–22^ and MPC-environment interactions.

## Future challenges

This Comment has discussed prospects and challenges for computational studies on monolayer-protected metal nanoclusters. Demand for functionalization and applications of these interesting nanomaterials creates a driving force to better understand interactions between MPCs and their environments where MPCs “operate”—for example, as catalysts, drug carriers, bioimaging contrast agents, as part of semiconducting nanomaterials or self-assembling bio-compatible plasmonic metamaterials. Computational investigation can greatly contribute to this understanding in the future, but it will require development of more efficient methods and new strategies, including hybrid methods that address problems across length- and timescales. For example, hybrid QM/MM methods, where part of the system is treated by quantum chemical methods and the environment is modeled by classical force fields or by coarse-grained methods, may be needed for optimizing nanocluster-drug-protein interactions to develop better targeted drug carriers in nanomedicine or for understanding the self-assembly of MPCs to design new materials. Similarly, the applicability of ML could be extended into complex MPC-environment interactions by combining it with DFT to find new efficient methods to solve complex catalytic reactions. Irrespective of the chosen strategy, emergence of new supercomputer platforms and graphics processing unit technology will require significant new algorithmic and software development work to harness the available computational power in full.
